# White Matter Lesions as Possible Predictors of Audiological Performance in Adults after Cochlear Implantation

**DOI:** 10.3390/brainsci11050600

**Published:** 2021-05-08

**Authors:** Steffen Knopke, Hans-Christian Bauknecht, Stefan Gräbel, Sophia Marie Häußler, Agnieszka J. Szczepek, Heidi Olze

**Affiliations:** 1 Department of Otorhinolaryngology, Head and Neck Surgery, Campus Virchow-Klinikum, Charité—Universitätsmedizin Berlin, 13353 Berlin, Germany; stefan.graebel@charite.de (S.G.); sophia-marie.haeussler@charite.de (S.M.H.); 2 Department of Neuroradiology, Campus Virchow-Klinikum, Charité—Universitätsmedizin Berlin, 13353 Berlin, Germany; christian.bauknecht@charite.de; 3 Department of Otorhinolaryngology, Head and Neck Surgery, Campus Charité Mitte, Charité—Universitätsmedizin Berlin, 10117 Berlin, Germany

**Keywords:** cochlear implantation, white matter lesions, Fazekas score, structural brain damage, predictive value, hearing ability

## Abstract

The presented prospective study investigated whether structural brain damage, measured with the Fazekas score, could predict hearing rehabilitation outcomes with cochlear implantation (CI). With a follow-up period of 24 months, this study included 49 bilaterally, postlingually hearing impaired CI candidates for unilateral CI (67.3 ± 8.7 years; 20 men, 29 women) older than 50 at the time of implantation. The differences in the predictive value between two age groups, 50–70 year-olds (mid-age; *n* = 26) and over 70-year-olds (elderly; *n* = 23), were analyzed. The patients were evaluated using speech perception (SP) measured in quiet (Freiburg monosyllabic test; FMT) and noise (Oldenburg sentence test; OLSA). The subjective hearing ability was assessed using Oldenburg inventory (OI). The Fazekas PVWM score predicted postoperative speech perception two years after CI in the mid-age population. The periventricular white matter lesions (PVWM) could explain 27.4% of the speech perception (FMT) variance. Our findings support the hypothesis about the influence of pre-existing WMLs on CI outcome. We recommend the evaluation of Fazekas score as a predictive factor for post-implantation hearing ability.

## 1. Introduction

The socio-demographic challenge of the 21st century includes the ongoing growth of the aging population. Unfortunately, extended life expectancy correlates with an increased prevalence of dementia and hearing impairment, the latter affecting 66% of people over 70 [1]. Hearing disorders are considered significant sensory health problems [2] and seem to accelerate the aging-dependent intellectual decline [3]. On the other hand, cognition is thought to have a control function that extends to speech intelligibility, demonstrated by an impairment of speech perception in background noise [4,5].

In 2017, the Lancet Commission on Dementia Prevention, Intervention and Care performed an extensive investigation of risk factors associated with dementia [6]. That work identified hearing loss as one of the leading preventable risk factors for cognitive dysfunction development. The model developed by the authors stratified the influenceable risk factors according to age clusters. While younger individuals mainly benefit from education, individuals in the mid-life stage of life (between 45 and 65), hearing loss was the most important preventable risk factor (9%) for dementia. According to this position paper, the older population’s profile of modifiable risk factors to dementia changes over the age of 65 due to smoking (5%), depression (4%), physical inactivity (3%), social isolation (2%), and diabetes (1%). In summary, the Lancet Commission’s report illustrates an individual approach to dealing with dementia syndrome by changing risk factors.

In light of current research findings, auditory rehabilitation is gaining importance in medical care, but despite that, according to the WHO [1], only around 17% of the hearing impaired undergo hearing rehabilitation. This depends on the type and location of the damage and ranges from fitting hearing aids to cochlear implants or even auditory brainstem implantation in selected cases. Cochlear implantation is an acknowledged auditory rehabilitation method for profound sensorineural hearing loss [1]. We have collected evidence about the positive impact of cochlear implantation on cognitive abilities [7,8], demonstrating that the positive effects of CI go far beyond the improvement of tonal hearing and speech perception. Given that hearing impairment affects health in various ways [9,10], its outcome might range from social isolation, depression, anxiety to the reduction in life quality [11,12,13]. We have demonstrated multiple positive effects of auditory rehabilitation with CI in younger and older people [11,13,14,15,16]. Another effect of hearing impairment is being discussed in the more recent scientific discussion. Structural brain damage also needs to be considered when studying the interactions between hearing impairment, aging, psychosocial aspects, and cognitive dysfunctions.

Lin et al. [17] demonstrated atrophy of hearing-relevant brain areas resulting from hearing impairment. Moreover, accumulating evidence supports the association between decreased cognitive stimulation (e.g., auditory stimulation) and cognitive dysfunction [2,18,19,20]. The question is whether the structural brain changes caused by the hearing loss negatively affect hearing rehabilitation. Apart from the duration of hearing impairment [21], other predictors for hearing rehabilitation are currently not applied in clinical practice [21,22]. It would be a milestone for patient counseling to identify a link between preventable neuronal damage and speech perception after CI.

Interestingly, not only the primary auditory cortex in the temporal lobe is affected by auditory deprivation but also the associated brain regions such as speech memory [23,24]. In elderly patients, the decrease in brain volume does not appear to be limited to specific areas. A reduction in total brain volume accompanied by hearing impairment has been reported, primarily due to damage to the white matter [25,26,27]. While the hearing abilities are examined audiologically and the cognitive abilities are evaluated using standardized test batteries, the structural brain damage requires imaging procedures.

Magnetic resonance imaging (MRI) can be used to evaluate brain volume and brain aging. This approach was used in current research evaluating hearing impairment and cognitive dysfunction [8,28,29,30]. Furthermore, MRI can identify the white matter lesions (WML), which correlate positively with age [31].

One particular cause of structural brain damage can be a small lesion of cerebral blood vessels [32], frequently seen in the aging population. White matter lesions may disrupt sensorimotor, behavioral, and cognitive networks leading to an impairment [33]. The classification of white matter lesions is based on the localization of cerebral vessels [25] and includes two groups: periventricular white matter lesions (PVWM) and deep white matter lesions (DWM). PVWMs correlate with the aging process (e.g., with cerebral hypotension, hypoperfusion, and atrophy), whereas DWMs correlate with the process of atherosclerosis or with endothelial inflammation [31]. The role of hearing impairment as a risk factor for the cerebral aging process has not been conclusively clarified [28]. Fazekas scoring is a method developed to quantify the degree of white matter lesions [25]. The Fazekas scores PVWM and DWM are added to a total score and enable a numerical estimate of the degree of damage to the entire brain.

The present study aimed to determine a predictive link between the Fazekas score and the outcome of hearing rehabilitation with a cochlear implant. The second aim was to determine possible differences in the Fazekas score predictive value between the mid-life and elderly populations.

## 2. Materials and Methods

The local Ethics Committee of Charité—Universitätsmedizin Berlin, corporate member of Freie Universität Berlin, Humboldt-Universität zu Berlin, and Berlin Institute of Health approved this prospective, non-interventional, longitudinal study (permit number EA2/030/13). All investigations were conducted according to the principles expressed in the Declaration of Helsinki. All patients gave their informed written consent. Forty-nine patients aged 50 years or older, 20 male and 29 female, were included in our study. The entire study population was divided into 50 to 70 years (mid-age), including 10 males and 16 females, and the second group of 70 years and older (elderly), including 10 males and 13 females. Detailed age-related patient characteristics are presented in Table 1. All subjects underwent unilateral cochlear implantation at the Charité Cochlear Implant Center of the Department of Otorhinolaryngology, Head and Neck Surgery, Berlin.

Patients were consecutively, prospectively enrolled in the study based on the following inclusion criteria:

- diagnosis of bilateral severe or profound hearing loss; speech perception ≤ 40% in the Freiburg monosyllabic test in quiet at 65 dB sound pressure level (SPL) with hearing aids of individually verified settings
- age over 18 years
- mother tongue German
- MRI-scan of the head and inner ear before surgery
- meeting of the clinical criteria for cochlear implantation:
  ○ post-lingual deafness (onset of hearing impairment after speech acquisition)
  ○ exclusion of disorders affecting the vestibulocochlear nerve (e.g., vestibular schwannoma)
  ○ unremarkable cochlear anatomy
  ○ possibility to use general anesthesia
  ○ the motivation for postoperative audiological rehabilitation

The data were collected between April 2009 and March 2016. The patients were referred to the hospital between April 2009 and March 2014 for unilateral cochlear implantation with a multichannel cochlear implant produced either by MED-El (Synchrony or Concerto, MED-El Innsbruck, Austria) or by Cochlear (Nucleus, Cochlear, Sydney, Australia). Before surgery, all patients underwent computed tomography (CT) and cranial magnetic resonance imaging (cMRI) of the temporal bone and the auditory nerve and central nervous system to exclude retro-cochlear disorders.

### 2.1. Radiological Assessment

Routine cMRI examinations before cochlear implantation were performed using a 1.5-T imaging system (Siemens Avanto, Siemens Medical Systems, Erlangen, Germany). A specific MRI imaging protocol for neurodegenerative disorders was applied. In detail, the MRI-sequences used for WML assessment consisted of axial PD/T2-weighted imaging (3340/14 milliseconds/3340/86 milliseconds, a field of view 23 cm, acquisition matrix 256 × 256 pixels, 3-mm section thickness), and coronal FLAIR imaging (9000/114 milliseconds, inversion time 2500 milliseconds, a field of view 23 cm, acquisition matrix 256 × 256 pixels, 4-mm section thickness).

To assess possible structural brain damage, a routine scoring of T2 hyper-intense white matter lesions visualized by MRI was conducted according to the Fazekas score [25]. The assessment was carried out during the primary MRI diagnosis by an experienced neuroradiologist (H.C.B.). The Fazekas scoring system grades and localizes the white matter lesions as follows:

Periventricular white matter lesions (PVWM)

0 = absent

1 = “caps” or pencil-thin lining

2 = smooth “halo”

3 = irregular periventricular signal extending into the deep white matter

Deep white matter lesions (DWM)

0 = absent

1 = punctate foci

2 = beginning confluence

3 = large confluent areas

**Figure 1:**
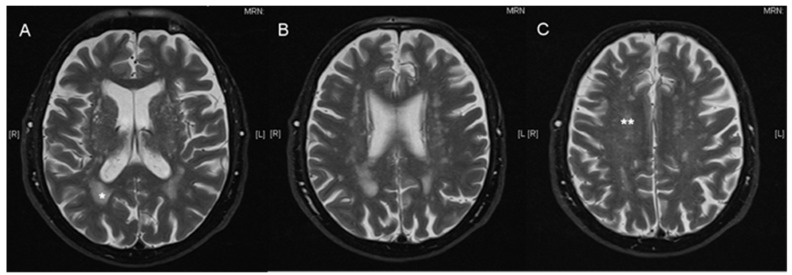
Exemplary MRI scan with annotated Fazekas score. Axial, T2 weighted cerebral MRI scan of an 87-year-old male patient before cochlear implantation with a Fazekas total score of 5, one asterisk indicates periventricular white matter lesions (PVWM *) score of 2; two asterisks indicate deep white matter lesions (DWM **) score of 3 (axial, **A**–**C**).

The Fazekas total score is a sum of the PVWM score and the DWM score (Fazekas total score = PVWM + DWM). Figure 1 demonstrates a representative T2 weighted cerebral MRI scan of an 87-year-old male patient with the marked Fazekas score.

The MRI scanning was performed before cochlear implantation. During the follow-up examinations at 6, 12, and 24 months post-surgery, audiological tests and subjective audiological assessment were conducted in parallel.

After the cochlear implantation, MRI was not performed because the implants were not suitable for magnetic resonance imaging, or the presence of an implant would result in an extinction artifact in the MRI image.

### 2.2. Audiological Performance: Speech Perception (SP)

The speech perception in quiet was assessed with the Freiburg monosyllabic test (FMT) at 65 dB SPL (sound pressure level). Before surgery, the test was performed with an individually fitted, conventional hearing aid, whereas after surgery, with the cochlear implant speech processor. The higher the measured value between 0% and 100%, the better the speech perception.

The Oldenburg sentence test (OLSA) measures the speech intelligibility threshold in background noise at 65 dB SPL. The standardized, broadband “Oldenburg noise” was used as background noise during the measurement, following previously described procedure [34]. Each sentence’s speech level was adjusted depending on the response to each test item to obtain the signal-to-noise ratio (*S*/*N*), at which the percentage of correct word score is 50% (critical *S*/*N*). In detail, twenty test sentences per list were presented in a random combination with a fixed scheme (name, verb, number, adjective, object). Two test runs were performed before the evaluation run. With a constant background noise level of 65 dB, the sound level was adapted stepwise to the number of correctly understood words. For sentences 2–5, the sound pressure level was adjusted by +3 and −3 dB depending on the percentage of words understood (0 to 5). For sentences 6 to 21, the sound pressure level was modulated by +/−2 dB. For the evaluation, the SPL used for sentences 12 to 21 were added and divided by 10. Finally, the background noise level was subtracted from this result in order to obtain the presented result (SVS).

The configuration S_0_N_0_ (speech and noise from the front) was used. The OLSA was used postoperatively with CI, as it enables an excellent approximation of everyday situations in background noise in rehabilitation. The reference value for persons with normal hearing is −7.1 ± 1.1 dB *S*/*N* (65 dB SPL), with a lower value indicating better speech perception [35].

### 2.3. Subjective Audiological Assessment: Oldenburg Inventory (OI)

For the evaluation of the subjective hearing ability, the OI questionnaire was used. The OI comprises a total score and three subcategories “hearing in quiet”, “hearing with background noise”, and “localization hearing”. There are 12 questions regarding everyday situations with scores from 1 to 5. The higher the score, the better the subjective hearing [36].

### 2.4. Statistical Analyses

For the statistical analyses, we used software SPSS version 25.0 (IBM, Ehningen, Germany). The results are shown as a mean ± standard deviation. The level of significance was 5%. Because of a lack of normal distribution in most of the dataset, Wilcoxon signed-rank test was used to compare the data before and after cochlear implantation. The statistical comparisons were made using the Mann-Whitney U-Test. Correlations were performed by computing the Spearman’s rank correlation coefficient (r_Sp_). The predictors of speech perception were identified using multiple linear regression with backward elimination. The requirements for implementation were checked. The test for the normal distribution of the residuals was performed using SPSS.

## 3. Results

### 3.1. Speech Perception (SP) Measured with the Freiburg Monosyllable Test (FMT)

**Figure 2:**
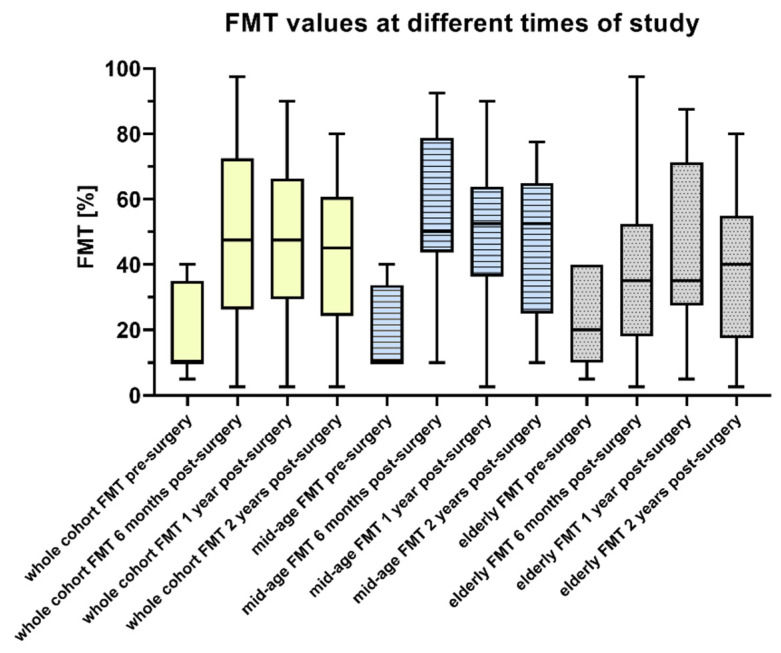
Speech perception measured with the Freiburg monosyllabic test (FMT). Box-and-whisker plots demonstrate the median values (box extend from the 25th to 75th percentiles) of FMT; whiskers indicate the minimum and maximum values. Shown is the FMT distribution in the whole cohort, mid-age (50–70) and elderly (>70) groups before surgery and 6, 12, and 24 months after surgery.

Prior to implantation, the mean speech perception of the whole cohort was 6.3% ± 12.1% (range: 0.0%–40.0%). Six months after implantation, it improved significantly (z = −5.393; *p* < 0.01) to 36.1% ± 31.3% in the entire cohort (Figure 2).

The whole cohort’s FMT score remained stable at one year (39.2% ± 28.2%; z = −0.824; *p* = 0.41) and two years (38.7% ± 24.2%; z = −0.272; *p* = 0.79) after cochlear implantation. The comparison between the groups, mid-age and elderly, indicated lack of significant difference (Table 2).

### 3.2. Speech Perception Measured by Oldenburg Sentence Test (OLSA)

Six months after implantation, the mean OLSA score in the entire cohort was 5.7 ± 5.00 dB *S*/*N*; one year after implantation 5.2 ± 7.0 dB *S*/*N*; two years after implantation 5.2 ± 9.6 dB *S*/*N*. There was a significant difference between the OLSA values obtained one and two years after implantation (*p* < 0.05). There was a significant difference in the OLSA scores obtained by the elderly group between 6 months and one year. However, the absolute difference between one year and two years was significantly greater. The value after two years was also associated with a significantly larger SD.

Comparing the mid-age and elderly groups revealed a lack of significant differences (Table 3).

### 3.3. Correlation Analysis between Speech Perception and Age

An age-group-specific rank correlation analysis was carried out to determine the degree of association between the participants’ age and speech perception. For 50 to 70-year-olds, there is no significant correlation (Table 4) between age and speech perception (OLSA and FMT) before the surgery and half a year, one year, and two years after the CI.

In the elderly group, no association between age and FMT could be determined (Table 5). In contrast, there was a significantly positive relationship between speech perception in noise (OLSA) two years post-surgery and age (r_Sp_ = 0.666; *p* < 0.01).

### 3.4. Subjective Audiological Assessment Using Oldenburg Inventory (OI)

The total score OI is a subjective assessment of the auditory abilities regarding hearing in quiet, hearing in noise, and sound localization. In the entire cohort, six months after implantation, the total score and the three subscales of OI improved significantly (OI total score: pre-surgery 2.02 ± 0.58 to 6 months post-surgery 2.91 ± 0.72; *p* < 0.01). When compared to the value before surgery, the improvement remained significant one year (OI total score 2.90 ± 0.67; *p* < 0.01) and two years after implantation (OI total score 2.86 ± 0.75; *p* < 0.01). A significant change in OI’s total score between half a year, one year, and two years after implantation was not found. The mid-age and elderly groups’ analysis also demonstrated significant improvement within all Oldenburg inventory domains six months after implantation (*p* < 0.01). Results remained stable over the whole follow-up period.

### 3.5. Radiological Assessment

In the entire cohort, the mean Fazekas total score in the cMRI before surgery was 1.2 ± 1.5, the mean PVWM score was 0.7 ± 0.9, and the mean DWM score was 0.5 ± 0.8. In the mid-age group, the Fazekas total score was 0.8 ± 1.2, whereas, in the elderly group, it was 1.7 ± 1.8 (Table 6). The difference in Fazekas scores between the mid-age and elderly was not significant (Fazekas total score z = −1.870, *p* = 0.06; PVWM z = −1.418, *p* = 0.16; DWM z = −1.738, *p* = 0.08), despite absolute differences in the mean values. In the entire study population, the Fazekas total score (r_Sp_ = 0.36; *p* < 0.05; 2-tailed) and the PVWM score (r_Sp_ = 0.34; *p* < 0.05; 2-tailed) correlate significantly with the patients’ age. No correlations were found between Fazekas scores and age in the mid-age and elderly groups (*p* > 0.05; 2-tailed).

### 3.6. Regression Analysis

Multiple linear regression with backward elimination was performed to examine the predictive value of Fazekas score concerning the post-implantation speech perception. When checking the basic requirements, the Fazekas total (the sum of PVWM and DWM), was excluded from further consideration due to a collinearity conflict. Thus, in addition to speech perception as a dependent variable, the variables PVWM, DWM, and age were examined as possible predictors for further consideration.

The distribution pattern was determined using a histogram of the residuals. There was a normal distribution of the predictor PVWM in the mid-age group concerning speech comprehension two years after implantation. A borderline normal distribution was determined for the OLSA two years after implantation in the elderly group concerning age. In contrast, the distribution of PVWM values regarding OLSA and FMT half a year after implantation was not normal in the elderly group. Alternative models were examined but could not be reliably calculated due to the small study population. Therefore, PVWM was excluded from the modeling regarding OLSA and FMT values half a year after implantation.

The regression analysis demonstrated that PVWM is a significant predictor for speech perception in quiet two years after CI for the mid-age group (Table 7). The PVWM could explain about 27.4% of the speech perception variance (FMT) two years after cochlear implantation. In the mid-age group, age was not a predictor for speech perception after implantation.

For the speech perception in noise (OLSA) in the elderly group, two years after implantation, the study participants’ age was a significant predictor (Table 8). The patient’s age can explain about 63.1% of the variance in the OLSA two years after CI. There was no significant model found for the Fazekas scores concerning speech perception in the elderly group.

## 4. Discussion

The present work examined whether structural brain damage measured with the Fazekas score is related to bilateral speech understanding of severely hearing-impaired adult individuals who underwent unilateral cochlear implantation. It was further investigated whether Fazekas score could be used as a factor predicting postoperative speech understanding.

To our best knowledge, this study demonstrates for the first time the age-dependent relationship between the Fazekas score PVWM and the speech perception in quiet after cochlear implantation in adults. White matter lesions measured with the Fazekas score were identified as predictors of postoperative speech perception in the mid-age group two years after cochlear implantation. The detailed analysis demonstrated that two years after CI, 27.4% of the speech perception in quiet (FMT) variance could be explained by the PVWM in the mid-age but not in the elderly. However, in the elderly group, a close relationship was seen between the patients’ age and the speech perception in noise. The age was analyzed in the multiple linear regression as a predictor for the OLSA. However, in the mid-age group, the age was not significantly associated with speech perception for either the FMT or the OLSA test scores.

Speech perception improved significantly after cochlear implantation in both age groups after just six months, corroborating the already published reports [12,16,37]. A stable plateau in speech restoration was seen using FMT for the outcome measurement 12 to 24 months after the implantation. Although no significant differences were found between the mid-age and the elderly groups regarding speech perception (FMT), a trend indicating the worse performance of the elderly group in FMT was seen. The lack of significance could be due to the data distribution connected with an insufficient number of participants. Concerning the OLSA test, there was a trend indicating steady improvement at each measurement point without statistical significance. A significant improvement from 0.5 years to one year after implantation was determined in the elderly group. A generally poor OLSA score two years after implantation among the elderly can be explained by this study’s already mentioned limitations (small sample). It could also be feasible that the elderly patients need an extended rehabilitation period or age-adopted rehabilitation techniques before achieving satisfactory results. Corroborating previously published research [38], the present study found no significant differences between the mid-age and the elderly concerning the audiological outcome after CI in the present study.

In addition to the audiological assessment, structural brain damage was measured with the Fazekas score. The regression analysis included the PVWM, DWM, and age as parameters potentially controlling speech perception. Fazekas et al. [25] observed that PVWM and DWM lesions can be found more often in older than younger adults and that the WML-free patients were younger than 70. The influence of WML on different brain functions, and in particular, on speech perception remains unclear. One could assume that a higher degree of white matter lesions associates with a decreased speech perception.

The cohort division into a mid-age and the elderly population has become an established procedure in recent research. Mid-life is usually defined as beginning around the age of 50. The transition to the elderly cluster occurs between 65 and 70 [6] and is based on primary research intelligence models. These models suggest a time-delayed decrease in fluid intelligence around the age of 50 and a decrease in crystalline intelligence around 70 [39,40]. The underlying structural brain lesions likely create the environment for the age-dependent, divergent influence of WMLs on speech perception in the mid-age and elderly CI-patient populations.

Various scoring systems are rating the WML, and the Fazekas scoring system is one of the commonly used ones. The neuroradiological Fazekas score was introduced for the MRI-based assessment of dementia and not exclusively to the potential damage to auditory brain regions. Consecutive neurological studies classified other cognitive functional disabilities and cerebral anomalies using MRI and the Fazekas score [41,42].

There are no published data concerning persons with bilateral, long-term, severe hearing impairment unilaterally implanted with CI and evaluated with the Fazekas score for white matter lesions to the best of our knowledge, making comparative analyses difficult. Valdés Hernández et al. [43] reported a Fazekas total score with a median of 2 in a cohort of individuals born in 1936 being on average 72.7 ± 0.7 years old at the time of MRI (mean ± SD). That cohort (*n* = 672) was not adjusted for specific diseases, and vascular risk factors were reported in 49% and a stroke in approximately 14% of the participants. Another cohort of 639 patients aged 65 to 84 years has been described in the LADIS study [44], evaluating the risk of developing physical dependence. The authors reported a mild Fazekas total score (0–2) in 44%, moderate (3–4) in 31%, and severe (5–6) in 25% of the participants. Hearing loss (not differentiated) was reported by 45.2% of the participants. Independent of the population-related comparison of the absolute Fazekas values, the Fazekas score’s reliability is shown in the description of the WML, with a high degree of correspondence between visual and semi-automatic methods.

Recent studies show that microstructural changes are already visible in the mid-life phase of life [2] and that structural changes are associated with hearing disorders [17]. In our study, the Fazekas score of the mid-age group was compared to the older group, but no significant difference between the groups was seen, possibly reflecting a small sample size. It remains to be determined why the Fazekas score increases with age. Known triggers of WML include vascular conditions and their consequences. Typically, such conditions increase with age, possibly leading to increased numbers of WML [31,44]. That would be a plausible explanation of our study’s findings, where age in the elderly group was a strong predictor for the OLSA. The triggers for organic brain changes lay primarily in the past, explaining the preliminary findings of the Lancet Commission on Dementia Prevention, Intervention and Care, which, in addition to hypertension and obesity, reported a hearing loss as the most significant risk factor for the later development of dementia in the mid-life phase [6]. Thus, we interpret our findings of PVWM predicting the postoperative bilateral speech perception two years after CI as an indication for the involvement of the hearing impairment in the development of the WMLs. The WMLs seem to have a predictive value on language rehabilitation in the mid-age but not in the elderly group.

The connection between hearing disorders and functional cognitive disorders was established in 1989 by Uhlmann et al. [19,45]. Current otoneurological research increasingly focuses on the auditory system’s aging [46] as a part of a general aging process [47]. A large amount of data on WML and its adverse effects on hearing function already exist for pediatric patients, especially after peri- or postpartum complications [48,49]. The WMLs occurring after congenital CMV infection were a significant trigger of hearing impairment in early childhood [7,50]. However, due to specific aspects of CMV infection [51,52,53], the CMV patients should be kept separately from other study cohorts when investigating the cause and effect of hearing impairments in adulthood.

Although complex medical and biomedical examinations became a part of adult hearing disorders’ elucidation, they mainly rule out other diseases and conditions. Predictors for hearing rehabilitation are not yet used in current clinical practice. Cochlear implantation’s clinical success is based on speech perception measured with various international tests lacking generally applicable criteria. Moreover, different parameters influencing the outcome of CI were reported. In addition to procedures presented in our study, using functional MRI has been suggested as an essential measure of the integrity of the central auditory pathway before implantation, predicting the CI outcome [54].

To date, apart from the acknowledged negative association between audiological performance and duration of deafness, no other standardized, recognized predictors are in clinical use [21,22]. At the same time, both mid-life and elderly benefit from hearing rehabilitation. The lack of hearing rehabilitation leads to a decrease in fluid intelligence due to an impairment of working memory. The decline in fluid intelligence already begins in the mid-life phase [39,55,56,57]. Simultaneously, in the elderly, social function and cardiovascular risk factors predominate as risk factors for cognitive decline. Depression and social isolation in the elderly are severe consequences of a lack of hearing rehabilitation [13,58,59,60] and essential preventable factors in later cognitive dysfunction [6,61]. To date, the mono-causal relationship between hearing impairment, structural brain damage, psychosocial dysfunction, and cognitive dysfunction is unclear. Likely, these factors realign instead of mutually influencing continuum that defines hearing impairment connected with WML as an avoidable factor in the mid-life phase. Unfortunately, presently, we cannot provide the answers to the questions regarding the potential connections. Diagnosing and treating risks delineated in the Lancet study should be an onset of a personalized, successful intervention.

Our study’s significant drawbacks are the small size of the study sample and the variation in the cohort, which makes it difficult to compare the age groups or stratify the results based on gender. Another limitation is an unequal distribution of patients regarding gender. Both of the limitations could be addressed in a more extensive, better-powered study, in which patients equally representing both genders and different age groups would be included. The study population itself introduces another limitation. A CI allows only a limited postoperative MRI examination. Frequent MRI monitoring to assess the progression of structural brain damage is therefore not possible or very limited.

## 5. Conclusions

The present study indicates the relationship between white matter lesions, hearing impairment, and WML’s possible effects on speech perception following cochlear implantation. The results also imply the influence of age or the limitations caused by aging with the consequence of structural brain damage in the elderly. However, in the mid-life phase, the connection between hearing impairment and speech perception seems to be partially accounted for by WML. Assuming the avoidable sensory deprivation as a trigger for WML, hearing rehabilitation is recommended for people with profound, bilateral hearing loss. With the CI, we have a tried and tested, and established method at our disposal. The extent to which the WML or its progress can be influenced by hearing rehabilitation remains withheld from future research. Further studies should confirm whether the Fazekas score can be used as a routine diagnostic marker to predict the outcome of hearing rehabilitation after cochlear implantation.

## Figures and Tables

**Table 1:** General patients’ characteristics.

	Whole Cohort		Age ≥ 50 to 70 Years		Age ≥ 70 Years	
[Years]	Mean	SD	Mean	SD	Mean	SD
Age at the point of cochlear implantation	67.27	8.69	61.10	6.37	74.25	4.77
Duration of hearing impairment	22.89	22.85	18.53	21.10	27.3	24.24

SD = standard deviation.

**Table 2:** Speech perception improves in the entire cohort and age subgroups after implantation. Speech perception in quiet was measured with the Freiburg monosyllabic test (FMT) at 65 dB SPL (sound pressure level). SD = standard deviation. “^1^” and “^2^” indicate significant differences between respective variables compared to their values per Wilcoxon signed-rank test. *p* ≤ 0.05.

	Whole Cohort		Age ≥ 50 to 70 Years		Age ≥ 70 Years	
	Mean	SD	Mean	SD	Mean	SD
pre-surgery	6.33 ^1^	12.07	5.96 ^1^	11.49	6.74 ^1^	12.93
post-surgery						
6 month	36.08 ^2^	31.29	39.17 ^2^	32.46	32.38 ^2^	30.23
12 month	39.22 ^2^	28.20	42.08 ^2^	27.32	35.95 ^2^	29.50
24 month	38.74 ^2^	24.20	41.76 ^2^	23.44	35.71 ^2^	25.15

**Table 3:** Distribution of speech perception in noise in patients groups. The mean speech perception threshold (dB *S*/*N*) was measured with OLSA test in 65 dB background noise. SD = standard deviation. “^1^” and “^2^” indicate significant differences between respective variables compared to their values per Wilcoxon signed-rank test. *p* ≤ 0.05.

	Whole Cohort		Age ≥ 50 to 70 Years		Age ≥ 70 Years	
Time Post-Surgery	Mean	SD	Mean	SD	Mean	SD
6 month	5.72	5.00	6.01	5.76	5.39 ^1^	4.09
12 month	5.17^1^	6.97	5.24	5.97	5.09 ^2^	8.25
24 month	5.21^2^	9.59	4.60	5.27	5.89	13.01

**Table 4:** Correlation analysis for speech perception and age in the mid-age group.

Age ≥ 50 and < 70 Years	FMT Pre-Surgery	FMT 0.5 Years Post-Surgery	FMT 1 Year Post-Surgery	FMT 2 Years Post-Surgery
Correlation coefficient (r_Sp_)	−0.010	0.090	−0.041	−0.074
sig. (2-tailed)	0.963	0.675	0.848	0.751
		OLSA 0.5 years post-surgery	OLSA 1 year post-surgery	OLSA 2 years post-surgery
Correlation coefficient (r_Sp_)		0.096	0.351	0.423
sig. (2-tailed)		0.664	0.101	0.071

**Table 5:** Correlation analysis for speech perception and age in the elderly group.

Age ≥ 70 Years	FMT Pre-Surgery	FMT 0.5 Years Post-Surgery	FMT 1 Year Post-Surgery	FMT 2 Years Post-Surgery
Correlation coefficient (r_Sp_)	−0.134	0.054	−0.003	0.115
sig. (2-tailed)	0.542	0.820	0.991	0.619
		OLSA 0.5 years post-surgery	OLSA 1 year post-surgery	OLSA 2 years post-surgery
Correlation coefficient (r_Sp_)		−0.046	0.233	0.666 **
sig. (2-tailed)		0.848	0.351	0.003

** *p* < 0.01.

**Table 6:** Distribution of Fazekas scores (total score; PVWM; DWM) in patients’ groups.

	Whole Cohort		Age ≥ 50 to 70 Years		Age ≥ 70 Years	
Pre-Surgery	Mean	SD	Mean	SD	Mean	SD
Total score	1.22	1.55	0.81	1.17	1.7	1.80
PVWM	0.73	0.93	0.50	0.76	1.0	1.04
DWM	0.49	0.79	0.31	0.55	0.7	0.97

**Table 7:** Multiple regression analysis with FMT two years after cochlear implantation in the mid-age group as a dependent variable. * *p* < 0.05; ** *p* < 0.01.

Variable	Unstandardized	Standardized	Standard Error
Constant	33.116 **		
PVWM	15.130 *	0.523 *	5.651
R^2^	0.274		
Adjusted R^2^	0.236		
F-Statistics (df 1;24)	7.168 *		

**Table 8:** Multiple regression analysis with OLSA 2 years after cochlear implantation in the elderly group as a dependent variable. ** *p* < 0.01.

Variable	Unstandardized	Standardized	Standard Error
Constant	−141.271 **		
Age	1.959 **	0.794 **	0.387
R^2^	0.631		
Adjusted R^2^	0.607		
F-Statistics (df 1;21)	25.675 **		

## Data Availability

Data are available upon request.
